# The inverse association between unhealthy eating habit and mucosal healing among patients with ulcerative colitis

**DOI:** 10.1186/s12876-021-01724-6

**Published:** 2021-04-07

**Authors:** Shinya Furukawa, Sen Yagi, Kana Shiraishi, Yu Hashimoto, Shogo Kitahata, Masakazu Hanayama, Kazuhiro Tange, Kenichiro Mori, Tomoyuki Ninomiya, Seiyuu Suzuki, Naozumi Shibata, Hidehiro Murakami, Katsuhisa Ohashi, Aki Hasebe, Hideomi Tomida, Yasunori Yamamoto, Eiji Takeshita, Yoshio Ikeda, Yoichi Hiasa

**Affiliations:** 1 Health Services Center, Ehime University, Matsuyama, Ehime Japan; 2 Department of Internal Medicine, Saiseikai Matsuyama Hospital, Matsuyama, Ehime Japan; 3 Department of Gastroenterology and Metabology, Ehime University Graduate School of Medicine, Ehime, Japan; 4 Department of Gastroenterology, Ehime Prefectural Central Hospital, Matsuyama, Ehime Japan; 5 Department of Gastroenterology, Sumitomo Besshi Hospital, Niihama, Ehime Japan; 6 Department of Gastroenterology, Ehime Prefectural Niihama Hospital, Niihama, Ehime Japan; 7 Ohashi Clinic Participating in Gastro-Enterology and Ano-Proctology, Niihama, Ehime, Japan; 8 Department of Gastroenterology, Shikoku Cancer Center, Matsuyama, Ehime Japan; 9 Department of Inflammatory Bowel Diseases and Therapeutics, Ehime University Graduate School of Medicine, Ehime, Japan; 10 Endoscopy Center, Ehime University Hospital, Ehime, Japan

**Keywords:** Eating quickly, Eating until full, Skipping breakfast, Eating habits, Ulcerative colitis

## Abstract

**Background:**

Although the association between eating habits which can be modified and digestive diseases has been reported, to date, no research has evaluated the association between eating habits and ulcerative colitis (UC). Thus, we investigate the association between eating behavior and clinical outcome in Japanese patients with UC.

**Methods:**

Eating quickly, eating until full, and skipping breakfast data was obtained from a self-administered questionnaire. Information on clinical outcome was collected from medical records. Mucosal healing (MH) and partial MH was defined as a Mayo endoscopic subscore of 0 or 0–1, respectively. Age, sex, BMI, current smoking, current drinking, prednisolone use, and anti-TNFα monoclonal antibody use were selected a priori as potential confounding factors.

**Results:**

Study subjects consisted of 294 Japanese patients with UC. Eating at speed moderate and eating quickly were independently inversely associated with MH: the adjusted odds ratios (ORs) were 0.38 (95% confidence interval [CI] 0.16–0.85) and 0.38 (95% CI 0.17–0.81) (*p* for trend = 0.033). Eating until full was independently inversely associated with MH: the adjusted OR was 0.38 (95% CI 0.27–0.86). MH in patients who skipped breakfast was marginally lower than that in patients who did not skip breakfast. No association between eating habits and clinical remission or partial MH was found.

**Conclusion:**

Among patients with UC, eating rate and eating until full may be independently inversely associated with MH but not clinical remission.

## Background

The prevalence of ulcerative colitis (UC) is on the rise, and it is widespread in different regions around the world [1, 2]. UC is a chronic inflammatory bowel disease (IBD) characterized by a disease course involving relapses and remissions [3]. Although a significant short-term benefit was observed after infliximab treatment, the colectomy rate remained stable in patients with UC. Despite many efforts, the surgical management of UC remains a significant challenge [4]. Mucosal healing (MH) is the key factor in quality of life, long-term remission, hospitalization, colorectal cancer, and surgery [5–8]. The therapeutic goal of UC is thus not only to relieve clinical remission, but also to achieve MH.

The association between eating habits and gastrointestinal diseases has been reported. The dinner-to-bedtime interval was inversely associated with gastroesophageal reflux disease (GERD) [9–13]. Eating quickly, eating until full, and skipping breakfast was positively associated with GERD [14]. Another study showed the positive relationship between skipping breakfast and severity of GERD [15]. Eating quickly and having irregular meal patterns were positively associated with functional dyspepsia (FD) [16–18] and constipation [19], respectively. Given the association between eating habits and gastrointestinal disease, we sought to examine if eating habits affect disease activity in patients with UC. To date, there have been no reports on the association between eating habits and UC. Thus, we aimed to evaluate the association between eating habits and clinical outcome in Japanese patients with UC.

## Material and methods

### Study population

Study subjects consisted of patients with UC who attended the Department of Gastroenterology and Metabology at the Ehime University Graduate School of Medicine or several affiliated hospitals in Ehime Prefecture (the Ehime Clinical Network for Alimentary Diseases study group). All patients were diagnosed with UC based on endoscopic, histological, radiological, and clinical criteria. All patients who were able to consent to this study and respond to the self-administered questionnaire were considered candidates for this study. This study was conducted in accordance with the Declaration of Helsinki. The institutional review board of the Ehime University Graduate School of Medicine approved the study protocol (#1505011). Trained staff obtained written informed consent from all enrolled patient**.** The study was registered at each hospital between 2015 and 2019.

### Measurements

Information on endoscopic findings, extent of UC, medication, and C-reactive protein (CRP) was collected from medical records. Each participant completed a self-reported questionnaire that collected data on eating habits. Current smoking was defined as positive if a subject reported smoking at least one cigarette per day. Current drinking was defined as positive if a subject reported any drinking, regardless of frequency or amount. Blood samples were taken in the morning after overnight fasting. Each patient’s BMI was calculated as their weight (kg) divided by the square of their height (m^2^). Blood examination was performed when a colonoscopy was booked or when the colonoscopy examination was performed, and there was up to two months between the two.

### Definition of eating habits

Eating rate, eating until full, and skipping breakfast data were determined from a self-administered questionnaire. Quick eating and slow eating were based, respectively, on whether a respondent answered “fast” or “very fast” and “slow” or “very slow” to the question, “How fast do you eat your meals?” (the 5 response options were very slow, slow, medium, fast, and very fast). Self-reported eating speed showed a high level of agreement with eating speed reported by a friend; the percentages of exact and adjunct categories of answers (e.g., very fast and fast were regarded as agreeing) were 46% and 47%, respectively [20]. Eating until full was defined on the basis of whether or not the respondent answered “Yes” to the question, “Do you tend to eat too much?” Skipping breakfast was defined as missing breakfast at least three times per week: “How often do you eat breakfast?” (the 6 response options were less than once a month, 1–3 times per month, 1–2 times per week, 3–4 times per week, 5–6 times per week, and every day).

### Definition of mucosal healing

The Mayo endoscopic subscore (MES) contains four categories [21]. Partial MH and MH were defined as category 0 and 0–1 in this study, respectively. A single endoscopic specialist was responsible for evaluating MES and MH, and was blinded to eating habits, CRP, and clinical remission.

### Statistical analysis

(1) Eating rate was divided into three categories: slow (reference), moderate and quick. (2) Eating until full was divided into two categories: no (reference) and yes. (3) Skipping breakfast was divided into two categories: no (reference) and yes. Estimations of crude odds ratios (ORs) and their 95% confidence intervals (CIs) for clinical remission, partial MH, and MH in relation to eating habits were performed using logistic regression analysis. Multiple logistic regression analyses were used to adjust for potential confounding factors. Age, sex, BMI, current smoking, current drinking, prednisolone use, and anti-TNFα monoclonal antibody use were selected a priori as potential confounding factors. Statistical analyses were performed using SAS software package version 9.4 (SAS Institute Inc., Cary, NC, USA). All probability values for statistical tests were two-tailed, and *p* < 0.05 was considered statistically significant.

## Results

Table 1 shows the clinical characteristics of study participants. A total of 294 Japanese patients with UC were included in this study. The percentages of men, current smoking, and current drinking were 59.2%, 7.5%, and 40.8%, respectively. Mean age, BMI, clinical remission, partial MH, MH, quick eating, eating until full, and skipping breakfast were 51.1 years, 22.75, 58.8%, 63.2%, 26.1%, 53.1%, 62.2%, and 13.6%, respectively. Most patients in the present study used 5-aminosalicylates.

**Table 1:** Clinical characteristics of the 294 study participants

Variable		n (%)
Age, years, mean ± SD		51.1 ± 16.2
Men (%)		174 (59.2)
BMI		22.75 ± 4.55
Disease extent (pancolitis/left-sided/proctitis/others)		122/83/82/7
Smoking		22 (7.5)
Drinking		120 (40.8)
Medication		
5-Aminosalicylates (%)		269 (91.5)
Prednisolone (%)		61 (20.8)
Azathioprine		47 (16.0)
TNF-α monoclonal antibody (%)		18 (6.1)
Clinical remission (%)		172 (58.8)
Mayo endoscopic subscore, mean ± SD		1.17 ± 0.9
Partial mucosal healing (Mayo endoscopic subscore ≤ 1) (%)		218 (63.2)
Mucosal healing (Mayo endoscopic subscore < 1) (%)		90 (26.1)
Eating behavior		
Eating speed		
Slow		40 (13.6)
Moderate		98 (33.3)
Quick		156 (53.1)
Eating until full		183 (62.2)
Skipping breakfast		40 (13.6)
CRP (mg/dl), mean ± SD		0.35 ± 0.79

*BMI* body mass index, *CRP* C-reactive protein, *SD* standard deviation, *TNF* tumor necrosis factor

Table 2 shows the crude and adjusted ORs and 95% CIs for clinical outcome in relation to eating rate. In the crude analysis, eating rate was not associated with clinical remission or partial MH. After adjustment for sex, age, BMI, current smoking, current drinking, and prednisolone and TNF-α monoclonal antibody use, eating moderately and eating quickly were independently inversely associated with MH; the adjusted ORs were 0.38 (95% CI 0.16–0.85) and 0.38 (95% CI 0.17–0.81), respectively (*p* for trend = 0.033).

**Table 2:** Crude and adjusted odds ratios and 95% confidence intervals for clinical outcome in relation to eating rate

Variable	Prevalence (%)	Crude OR (95% CI)	Adjusted OR (95% CI)
Eating rate			
Clinical remission			
Slow	26/40 (65.0)	1.00	1.00
Moderate	59/98 (60.2)	0.82 (0.37–1.73)	0.70 (0.30–1.59)
Quick	87/156 (55.8)	0.68 (0.32–1.38)	0.67 (0.30–1.46)
* p* for trend			0.39
Partial MH (MES ≤ 1)			
Slow	28/40 (70.0)	1.00	1.00
Moderate	59/98 (60.2)	0.65 (0.29–1.40)	0.61(0.26–1.38)
Quick	100/156 (64.1)	0.77 (0.35–1.59)	0.74 (0.32–1.61)
* p* for trend			0.74
MH (MES < 1)			
Slow	17/40 (42.5)	1.00	1.00
Moderate	25/98 (25.5)	0.46 (0.21–1.01)	0.38 (0.16–0.85)
Quick	35/156 (22.4)	0.39 (0.19–0.82)	0.38 (0.17–0.81)
* p* for trend			0.038

Odds ratios were adjusted for age, sex, body mass index, current smoking, current drinking, prednisolone use, and TNF-α monoclonal antibody use

*CI* confidence interval, *MES* Mayo endoscopic subscore, *MH* mucosal healing, *OR* odds ratio

Table 3 shows the association between eating until full and clinical outcome. Eating until full was independently inversely associated with only MH after adjustment for confounding factors: the adjusted OR was 0.38 (95% CI 0.27–0.86).

**Table 3:** Crude and adjusted odds ratios and 95% confidence intervals for clinical outcome in relation to eating until full

Variable	Prevalence (%)	Crude OR (95% CI)	Adjusted OR (95% CI)
Clinical remission			
No	71/111 (64.0)	1.00	1.00
Yes	101/183 (55.2)	0.69 (0.43–1.12)	0.80 (0.46–1.38)
Partial MH (MES ≤ 1)			
No	75/111 (67.6)	1.00	1.00
Yes	112/183 (61.2)	0.76 (0.46–1.24)	0.67 (0.38–1.16)
MH (MES < 1)			
No	40/111 (36.0)	1.00	1.00
Yes	37/183 (20.2)	0.45 (0.26–0.76)	0.48 (0.27–0.86)

Odds ratios were adjusted for age, sex, body mass index, smoking, drinking, prednisolone use, and TNF-α monoclonal antibody use

*CI* confidence interval, *MES* Mayo endoscopic subscore, *MH* mucosal healing, *OR* odds ratio

The association between skipping breakfast and clinical remission is shown in Table 4. The percentage of MH in patients who skip breakfast was marginally lower than in those who did not skip breakfast (*p* = 0.06). Skipping breakfast was not, however, associated with all clinical outcomes after adjustment for confounding factors.

**Table 4:** Crude and adjusted odds ratios and 95% confidence intervals for clinical outcome in relation to skipping breakfast

Variable	Prevalence (%)	Crude OR (95% CI)	Adjusted OR (95% CI)
Clinical remission			
No	147/254 (57.9)	1.00	1.00
Yes	25/40 (62.5)	1.21 (0.62–2.46)	1.40 (0.66–3.06)
Partial MH (MES ≤ 1)			
No	160/254 (63.0)	1.00	1.00
Yes	27/40 (67.5)	1.22 (0.61–2.55)	1.14 (0.54–2.50)
MH (MES < 1)			
No	71/254 (30.0)	1.00	1.00
Yes	6/40 (15.0)	0.46 (0.17–1.06)	0.52 (0.18–1.26)

Odds ratios were adjusted for age, sex, body mass index, smoking, drinking, prednisolone use, and TNF-α monoclonal antibody use

*CI* confidence interval, *MES* Mayo endoscopic subscore, *MH* mucosal healing, *OR* odds ratio

## Discussion

In the present study, eating rate and eating until full but not skipping breakfast were independently inversely associated with MH among 294 Japanese patients with UC. Eating habits were not associated with clinical remission or partial MH. This is the first study to report the association between eating habits and clinical outcome in patients with UC.

The association between eating habits and the onset of lifestyle-related diseases has previously been examined. Eating quickly was associated with obesity [22–25] and blood pressure [22–24]. Eating until full was associated with obesity and central obesity [26, 27]. Skipping breakfast was positively associated with obesity [28], low-density lipoprotein cholesterol [29], and onset of diabetes [30].

The association between eating habits and digestive diseases has also been reported. Eating dinner late was associated with GERD. In a case–control study of Japanese patients, the dinner-to-bedtime interval was independently inversely associated with GERD [9]. Similar results were reported in Iranian [10], Albanian [11], Korean [12], and a Japanese study [13]. Eating quickly is associated with GERD and FD. Eating quickly was positively associated with GERD in a case–control study of 1518 Chinese subjects [14]. In a cross-sectional Iranian study of 4763 subjects, eating quickly was associated with FD [16]. In a Serbian study, the statistical tendency to eat quickly among patients with FD was high [17]. In a Korean study of 87 patients, the frequency of eating meals within 13 min among patients with FD was higher than that among patients with GERD and the control group [18].

Eating until full and skipping breakfast were positively associated with GERD in the above-mentioned Chinese study [14]. In a cross-sectional Japanese study in which 19,864 subjects underwent medical checkups, skipping breakfast had a positive relationship with GERD severity based on a frequency scale for symptoms of GERD [15]. Skipping meals, including skipping breakfast, was positively associated with FD [16, 17, 31]. Skipping breakfast was positively associated with constipation in Japanese university students [19]. In contrast, no association between skipping breakfast and GERD was found in a Turkish study [32] and another Japanese study [33]. Several studies found a positive association between unhealthy eating behaviors and digestive diseases. The association between eating behavior and digestive diseases remains inconsistent, however. The inconsistency might be explained at least in part by sample size, age, BMI, eating behavior definitions, and questionnaire differences. To the best of our knowledge, no studies have investigated the association between eating habits and IBD, including Crohn’s disease. The underlying mechanism linking eating habits and MH among patients with UC remains unclear. Eating rate is inversely associated with numbers of chewing. Chewing was associated with mucosal blood flow via nitric oxide [34]. Eating until full might affect the ghrelin and leptin levels. In IBD model mice, both ghrelin and leptin were associated with intestinal motility and inflammation [35, 36]. Higher ghrelin and lower leptin in patients with UC had been observed compared to healthy controls [37]. Thus, eating quickly might induce mucosal ischemia via low nitric oxide, and eating until full might activate intestinal motility and inflammation via gastrointestinal hormones.

Our study has a few limitations. First, this was a cross-sectional study. Eating habits can, however, undergo intervention. Further research is needed to confirm the association between eating habits and MH in the future. Second, most of the patients were receiving treatment. The long duration of such treatment might have affected eating habits and clinical outcomes. Third, potential confounding factors, such as dietary patterns, nutritional information for the specific foods consumed, and other health-related behaviors, might be missing. Fourth, although self-reported eating rate was validated in a previous study [18, 20], information on eating habits was based on a self-reported questionnaire. Data on frequency of skipping breakfast and eating until full were also based on the responses to a self-reported questionnaire. Fifth, this cohort consisted only of Japanese patients with UC. As dietary patterns vary among cultures, further research is needed to confirm the association between eating habits and MH in UC patients from other parts of the world. Finally, the patients of the present study were not likely representative of patients with UC in Japan. Nevertheless, the men: women ratio, median age, and use of biologics, prednisolone, 5-aminosalicylates, and thiopurines were similar between the present study (59.2%, 48.0 years, 6.1%, 20.8%, 91.5%, and 16.0%, and, respectively) and a Japanese national study based on UC claims data in 2016 (63.9%, 44.0 years, 9.0%, 15.5%, 96.2%, and 13.8%, respectively) [38].

## Conclusions

Eating rate and eating until full but not skipping breakfast may be independently inversely associated with MH among Japanese patients with UC.

## Data Availability

The datasets used and/or analyzed during the current study are available from the corresponding author on reasonable request.

## Abbreviations

BMI: Body mass index
CRP: C-reactive protein
GERD: Gastroesophageal reflux disease
IBD: Inflammatory bowel disease
MES: Mayo endoscopic subscore
MH: Mucosal healing
TNF: Tumor necrosis factor
UC: Ulcerative colitis
